# Oriented Interpenetrating Capillary Network with Surface Engineering by Porous ZnO from Wood for Membrane Emulsification

**DOI:** 10.3390/ma17092113

**Published:** 2024-04-30

**Authors:** Yaodong Chen, Xiaolin Liu, Gonggang Liu, Shanshan Chang, Jinbo Hu

**Affiliations:** 1 Hunan Province Key Laboratory of Materials Surface & Interface Science and Technology, College of Materials Science and Engineering, Central South University of Forestry and Technology, Changsha 410004, China; cyd222610@163.com (Y.C.); changelxy@hotmail.com (S.C.); 2 Hunan Lintec Co., Ltd., Changsha 410600, China; liuxiaolin@lintec-x.com

**Keywords:** membrane emulsification, wood, emulsion, ZnO nanoparticles, microfluid

## Abstract

Membrane emulsification technology has garnered increasing interest in emulsion preparation due to controllable droplet size, narrower droplet size distribution, low energy consumption, simple process design and excellent reproducibility. Nevertheless, the pore structure and surface engineering in membrane materials design play a crucial role in achieving high-quality emulsions with high throughput simultaneously. In this work, an oriented interpenetrating capillary network composed of highly aligned and interconnected wood cell lumens has been utilized to fabricate an emulsion membrane. A novel honeycomb porous ZnO layer obtained by a seed prefabrication–hydrothermal growth method was designed to reconstruct wood channel surfaces for enhanced microfluid mixing. The results show that through the unique capillary mesh microstructure of wood, the emulsion droplets were smaller in size, had narrower pore-size distribution, and were easy to obtain under high throughput conditions. Meanwhile, a well-designed ZnO layer could further improve the emulsion quality of a wood membrane, while the emulsifying throughput is still maintained at a higher level. This demonstrates that the convection process of the microfluid in these wood capillary channels was intensified markedly. This study not only develops advanced membrane materials in emulsion preparation, but also introduces a brand-new field for functional applications of wood.

## 1. Introduction

Emulsification technology plays a crucial role in various industries and fields, such as food and beverages, pharmaceuticals, cosmetics, and petroleum [1,2,3,4,5]. It involves the process of mixing two immiscible liquids, such as oil and water, to form a stable emulsion. The ability to efficiently create and stabilize emulsions is essential for the production of countless products and has a significant impact on their quality and functionality [6,7]. Researchers have developed innovative emulsification techniques, such as ultrasonic emulsification, high-pressure homogenization, and microfluidics, which have improved the efficiency and stability of emulsion formations [8,9,10]. Membrane emulsification is a versatile technique and has been widely used for producing emulsions due to its irreplaceable advantages in tunable emulsion size distribution and low energy consumption compared to conventional emulsification methods [11,12,13,14,15]. It also offers better control over the emulsification process, allowing for the production of stable emulsions with enhanced properties [16,17]. Moreover, membrane emulsification has also been demonstrated to be effective in preparing uniform micro-/nanoparticles with narrow size distributions and more complicated nanostructures [18,19,20,21]. It is a versatile and promising technique for the preparation of monodispersed particles with tailored properties for various applications in fields such as drug delivery, catalysis, and materials science.

To realize its ultimate commercial application, membranes for emulsification should have narrow pore-size distribution, low resistance to flow, excellent mechanical and chemical durability, high thermal resistance, wettability that can easily be modified, and should be biocompatible, sterilisable and cheap [22,23,24,25]. Most importantly, a high-quality emulsion with high throughput is an intractable issue for membrane emulsification due to a trade-off effect which is ubiquitous in other membrane technologies [23,26,27]. The trade-off in membrane emulsification lies in finding the balance between the desired droplet size and production rate. Smaller droplets offer increased stability and a larger interfacial area, which is desirable for certain applications such as drug delivery systems and food products. However, the production rate decreases as the droplet size decreases. This trade-off can be optimized by pore structure design and surface engineering of membrane materials to achieve the desired emulsion characteristics [28,29,30]. While the sinuous pore structure of most membrane materials promotes fluid mixing, it also comes with drawbacks such as higher pressure drops and decreasing flux [31,32]. Hence, suitable pore structure should be considered when selecting a membrane material for emulsification application, weighing the benefits of fluid mixing against the trade-offs in performance.

As a kind of renewable and green biomass material, wood has been widely used for centuries in construction, furniture making, and energy production [33,34,35]. It is primarily composed of cellulose, hemicellulose, and lignin. Among these three components, cellulose serves as the basic unit of wood cell walls, accounting for 40–50% of wood’s composition, while lignin comprises 20–30%, and hemicellulose contains 15–30% [35]. Recently, wood is frequently used to design monolithic functional materials on account of its unique porous structure, decent mechanical and chemical durability, good modifiability, low cost, excellent hydrophilicity, and ease of processing, making it attractive for a wide range of applications in energy, environment, catalysis, and other fields [36,37,38,39,40]. Notably, the porous structure of wood consists of numerous well-aligned long lumens interconnected by pits and ray cells, forming an oriented interpenetrating capillary network [41]. In this way, nutrients for tree growth can be rapidly transported in the direction of growth, thus facilitating effective mass transfer. Many studies have demonstrated that utilizing the pore structure of wood along its growth direction enables a highly efficient mass transfer in water treatment [42,43,44,45]. In Hu’s research [46], a basswood matrix decorated with Pd nanoparticles was used to degrade methylene blue (MB), taking full advantage of the basswood structure along its growth direction. Owing to the developed mass transfer system, the water treatment rate of the wood membrane can reach up to 1 × 10^5^ L·m^−2^·h^−1^ with a high MB removal efficiency (>99.8%) concurrently, displaying high water treatment efficiency. In view of the above analysis, wood is an ideal emulgator to produce high-quality emulsions with high throughput. But so far there has been no literature reported on the application of wood in membrane emulsification. On the other hand, ZnO and its related compounds, which are hydrophilic and rich in hydroxyl groups, have been widely used to construct micro/nano roughnesses for designing composites due to their low cost, ease of generation, and environmental friendliness [47,48,49].

In this work, wood with unique hierarchical porosity was utilized to fabricate emulsion membranes. A novel honeycomb porous ZnO layer was designed to further modify the wood channel surface by a seed prefabrication–hydrothermal growth method. An oriented interpenetrating capillary network composed of highly aligned and interconnected wood cell lumens was utilized as a microfluid transfer system. Highly aligned wood cell lumens provide high throughput, and interconnected channels between adjacent cell lumens strengthen mixed convection processes of microfluids. A well-designed ZnO layer with improved lipophobicity and a convex groove structure on a wood channel surface could further promote mixed convection to form high-quality oil-to-water emulsions. As a proof of concept, the emulsification properties of these prepared membranes were studied, and the convection processes of the microfluid in these wood capillary channels were also discussed. This study not only contributes to the development of advanced membrane materials for emulsion preparation, but also pioneers a brand-new field for wood applications. It showcases the transformative potential of these innovative materials in improving industrial processes and expanding the scope of wood utilization.

## 2. Materials and Methods

### 2.1. Materials

Poplar and fir wood came from a forest in China and was cut into slices with a diameter of 25 mm and the different thicknesses required for the experiment. Soybean oil was provided by the Jiusan Grain and Oil Industry Group Co., Ltd. (Harbin, China); anhydrous zinc acetate, sodium hydroxide and sodium sulfite by the Shanghai Titan Technology Co., Ltd. (Shanghai, China); urea and Tween 80 by the Sinopsin Chemical Reagent Co., Ltd. (Shanghai, China); and deionized water by the laboratory.

### 2.2. Preparation of ZnO-Wood Membrane

Poplar wood slices were put into a mixed solution of 0.2 mol/L sodium sulfite and sodium hydroxide (molar ratio of 1:1) and washed several times with deionized water to remove impurities and some of the lignin to obtain a smooth pore structure. Then, they were soaked in ethanol to replace the solvents and subsequently immersed in a 1 mol/L zinc acetate/ethanol solution, where it was stirred for two days. The zinc acetate solution was then heated at 60 °C with dropwise addition of 1 mol/L NaOH (in ethanol). When the solution began to turn from transparent to translucent, the addition of NaOH was stopped and the solution was heated for another 10 min to make a ZnO nanosol. It was then immersed in the zinc oxide nanosol for 24 h before removal. Subsequently, zinc acetate and urea were dissolved in deionized water at a total concentration of 0.02 mol/L (1:2 molar ratio of zinc acetate to urea). The treated wood slice was transferred into a hydrothermal reactor with a zinc acetate and urea solution and heated at 90 °C for a certain time (2 h, 6 h, 10 h). Finally, the sample was removed from the growth solution, rinsed multiple times with ethanol and deionized water, and dried at 60 °C for 12 h. The obtained sample was denoted as ZnO-wood.

### 2.3. Characterization

A scanning electron microscope (Sigma 300, Carl Zeiss AG, Oberkochen, Germany) was used to observe the morphology of wood membrane samples, and the element distributions of the samples were analyzed by SEM-EDS. An optical contact angle tester (JGW-360B, ZDYQ, Chengde, China) was used to verify the hydrophilicity of the samples. At room temperature, droplets (0.3 μL) were dropped on the surface of the membrane for a few seconds. Then, the value of the contact angle was recorded by the algorithm program. Each sample was measured five times at different points and averaged. When testing the oil contact angle in water by using a contact-angle measuring instrument (LSA-100, LAUDA Scientific GmbH, Königshofen, Germany), the sample is first wetted with deionized water and drops of oil (0.3 μL) inside the deionized water on the membrane surface for a few seconds. Similarly, the value of the contact angle is recorded by the algorithm program. A synchronous thermal analyzer (STA 6000, PerkinElmer, Hopkinton, MA, USA) was used to test the thermal stability of the sample in an argon atmosphere at a heating rate of 5 °C·min^−1^, with a temperature range of 30 to 800 °C. To obtain the mass content of ZnO, the calcine atmosphere was changed into oxygen. An X-ray diffractometer (Smartlab SE, Rigaku, Tokyo, Japan) was used to analyze the phase structure and crystallinity of the sample. A Fourier infrared spectrometer (Nicolet iS5, Thermo Fisher Scientific, Waltham, MA, USA) was used to test chemical bond changes in samples in the wave number range of 400 to 4000 cm^−1^. Images of oil-in-water emulsions were obtained using an optical microscope (H550S, Nikon, Tokyo, Japan) and the sizes of the dispersed-phase droplets in a continuous phase were determined by statistical analysis. The statistical data of droplet size were determined using the image processing software Nano Measure (version 1.2.0). For each experiment, at least 500 droplets were measured for representative dimensional counts. Moreover, to further accurately assess the droplet size of the obtained oil-in-water emulsions and their distribution, a Malvern Zetasizer (Zetasizer Nano ZS, Malvern Instruments, Almelo, UK) was also adopted.

### 2.4. Membrane Emulsification Experiment

In this experiment, a homemade device was used to produce emulsions, and a small air compressor with a surge tank provided membrane pressure. Deionized water was used as the continuous phase and soybean oil was used as the separate phase. The oil to water ratio was 9:1, and T-80 was added into the mixture as emulsifier (the ratio of emulsifier to the two phases is 1:50). Then, the wood membrane samples were fixed and encapsulated in the homemade device by fastening a flange with a rubber seal ring. Next, the prepared mixture was added to the chamber connected to the air pressure inlet, and it was forced to pass through the membrane samples at a pressure of 0.1 bar to achieve an emulsion in the other chamber. The flux was measured as follows:

(1) $$J=\frac{V}{At}$$

where J is the emulsion flux (L·m^−2^·h^−1^), V is the total amount of emulsion collected (L), A is the effective membrane area (m^2^), and t is the total emulsion time (h).

## 3. Results and Discussion

**Figure 1:**
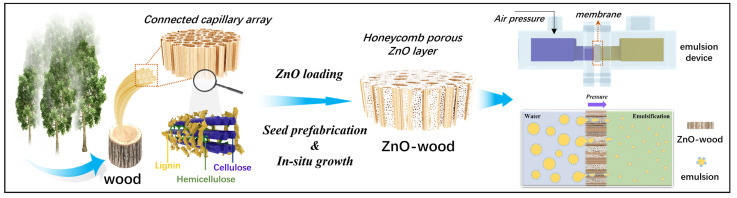
Strategy of constructing ZnO-wood and its membrane emulsification process.

The pore structure and surface wettability regulation of the membrane have a strong impact on the emulsification performance of the membrane. A rough surface structure and reduction in membrane pore size is conducive to the convective mixing of microfluid [23,24,50]. As for oil-in-water emulsions, the membrane needs to be highly hydrophilic or lipophilic. In this way, the aqueous phase can easily wet the surface of the membrane and form a continuous film, thus allowing the dispersed phase to pass through the membrane drop by drop. Therefore, to obtain high-quality oil-in-water emulsions, ZnO loading on a wood capillary channel surface was used to further adjust the pore structure and surface hydrophilicity of wood. The strategy of constructing ZnO-wood and its membrane emulsification process are shown in Figure 1. To overcome the growth resistance in capillary space and obtained uniformly loading ZnO nanoparticles, seed prefabrication combined with hydrothermal growth was used. Here, poplar as a kind of hard wood was used as raw material to prepare ZnO-wood.

**Figure 2:**
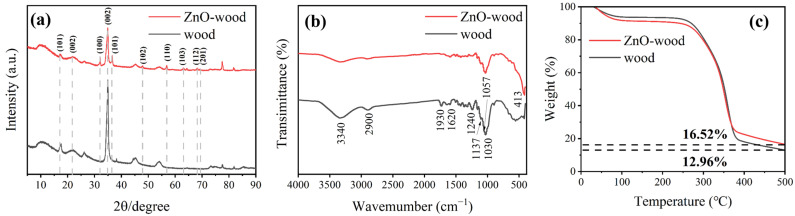
(**a**) XRD patterns, (**b**) FTIR spectra, (**c**) TG curves of wood and ZnO-wood.

Figure 2a shows XRD patterns of poplar and ZnO-wood. The XRD peaks at approximately 2θ = 15.8°, 22°, and 34.5° correspond to the crystal faces (101), (002), and (040) of cellulose crystal from poplar, respectively [51]. For ZnO-wood, the hexagonal wurtzite phase of ZnO (JCPDS: 99-0111) can be observed clearly, suggesting the successful loading of ZnO nanoparticles. The FTIR spectra of poplar and ZnO-wood are displayed in Figure 2b. The primary absorption peak of poplar is located around 3340 cm^−1^, which corresponds to the contraction vibration of the free hydroxyl group and intramolecular hydrogen bonds. Another absorption peak is observed at 2900 cm^−1^, attributed to the contraction vibration of the methyl group and methylene group. Additionally, there is an absorption peak at 1930 cm^−1^, which can be attributed to the vibration peak of the carbonyl stretching (C=O). The vicinity of 1620 cm^−1^ corresponds to the skeletal vibration of the benzene ring in lignin [46]. Another absorption peak at 1240 cm^−1^ is associated with the contraction vibration of the -CH stretching. Furthermore, the peaks observed near 1057 cm^−1^, 1039 cm^−1^, and 1137 cm^−1^ correspond to the C-O-C stretching vibration, C-O stretching vibration, and C-H vibration, respectively, associated with both cellulose and hemicellulose [52]. However, these peaks significantly weaken for ZnO-wood, which may be due to the coverage of the loaded ZnO layer. Moreover, a strong absorption peak corresponding to the contraction vibration of the Zn-O bond could be observed at 413 cm^−1^ [53], indicating the successful loading of zinc oxide onto the poplar. The thermal stability of the membrane ensures that the emulsification process operates at higher temperatures. To investigate the thermal stability of the membrane samples, the TG curves of poplar wood and ZnO-wood in an argon atmosphere are provided, as depicted in Figure 2c. This indicates that both poplar and ZnO-wood show decent thermal stability under 250 °C, which is superior to most organic membrane materials. However, there is a rapid pyrolysis process from 300–400 °C. During the whole thermal decomposition process of poplar wood, the main components such as cellulose, hemicellulose, and lignin begin to decompose when the temperature exceeds 250 °C, releasing water and volatile organic compounds. As the temperature rises further to between 300 °C and 400 °C, the wood undergoes a more pronounced decomposition process. Carbohydrates, cellulose, and hemicellulose decompose further, releasing a higher amount of volatile organic compounds and smoke. When the temperature surpasses 400 °C, the wood undergoes carbonization, where lignin and other organic matter are converted into carbonaceous residues, while water and volatile organic compounds are completely released [54]. On the other hand, in order to determine the average content of zinc oxide nanoparticles in the ZnO-wood, thermogravimetric degradation in an oxygen atmosphere was also conducted. The results in Figure S1a demonstrate that the mass content of zinc oxide nanoparticles in the wood is 1.23%. Moreover, thermal treatment and organic solvent resistance on the dimensional stability of ZnO-wood were also studied, as shown in Figure S1b. The wood dimensions remained almost unchanged during heat treatment at 120 °C and N-Methylpyrrolidone soaking, suggesting a wider operating environment for the membrane emulsification process.

**Figure 3:**
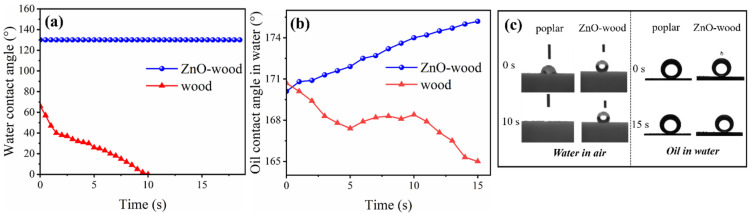
(**a**–**c**) Surface wettability test of poplar and ZnO-wood.

Surface wettability is a crucial factor in emulsification properties of the membrane [15]. Figure 3a illustrates the water contact angles of poplar and ZnO-wood. The result shows the initial water-contact angle of poplar is around 65°. After 10 s, water droplets gradually infiltrate into the interior of the poplar, indicating that poplar exhibits good hydrophilicity. It is due to the presence of hydroxyl (-OH) groups in the wood’s chemical structure, which can form hydrogen bonds with water molecules [55]. However, the water contact angle of the ZnO-wood was about 130° higher than that of the virgin wood, which may be due to the rough structure of the ZnO nanoparticles on the wood surface. To further test its real surface wettability and to exclude the effect of the coarse structure of the ZnO, the oil contact angles of poplar and ZnO-wood in water were also investigated. Figure 3b presents the oil contact angles in water, and Figure 3c shows the contact angle of water in air and oil in water at different times. The results show that ZnO-wood has excellent lipophobicity, superior to poplar. This indicates that ZnO loading could enhance its lipophobicity or hydrophilicity, which inhibits the formation of a continuous oil film and causes the dispersed phase to generate an oil-in-water emulsion.

**Figure 4:**
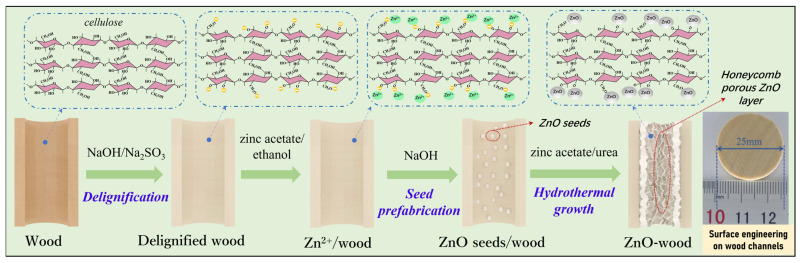
ZnO-wood preparation process and its mechanism.

**Figure 5:**
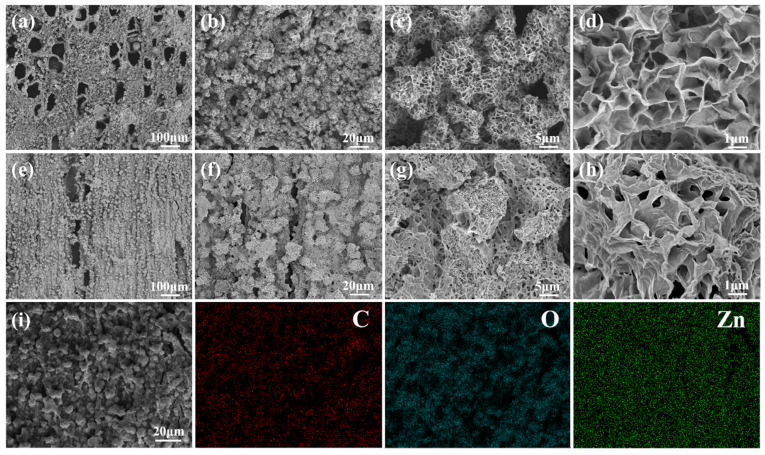
SEM images of ZnO-wood in (**a**–**d**) cross-section and (**e**–**h**) growth direction; (**i**) elemental mappings (C, O, Zn) in the EDS spectrum in cross-section.

The preparation process of ZnO-wood and its mechanism are illustrated in Figure 4. First, the wood slice was treated by a mixed NaOH/Na_2_SO_3_ solution to remove impurities and some lignin from the wood slices to obtain a permeable pore structure. Meanwhile, an electronegative wood surface was obtained by alkali treatment. This surface can provide abundant adsorption sites for Zn^2+^, to prepare ZnO seed crystal on the surface of wood cell walls. During the hydrothermal process, these ZnO seed crystal could further induce ZnO crystal growth, forming uniform ZnO nanoparticles with unique morphology in the wood capillary network. The morphologies of growing ZnO nanoparticles from ZnO-wood are shown in Figure 5a–h. From the SEM images of the ZnO-wood cross-section, the novel honeycomb-like porous ZnO layer on the wood channel surface can be easily observed. It consists of sinuate and connected ZnO nanosheets with a thickness of about 20 nm. The SEM images of dissected wood cell display a similar porous ZnO layer, but the difference is that more corrugations appear in these ZnO nanosheets. Though wood cell lumens are full of honeycomb porous ZnO layers, it can be noticed that the wood pores remain unobstructed. In addition, the element mapping of ZnO-wood (Figure 5i) shows C, O, and Zn elements are uniformly distributed on the surface of wood cell lumens. To investigate the effect of hydrothermal time on the growth of ZnO layers, the morphologies and structures of ZnO-wood with various hydrothermal times are observed in Figure S2. This indicates there no ZnO nanosheets exist on the wood framework at 2 h hydrothermal time. With the hydrothermal time increasing to 6 h, an obvious honeycomb porous ZnO layer with ZnO nanosheets forms. Under the condition of 10 h hydrothermal time, the structure of the ZnO layer is more regular and compact, and these ZnO nanosheets have a better engagement. Additionally, with the increase in hydrothermal time, the results of element mapping and the EDS spectrum demonstrated that the ZnO nanoparticles were homogeneously distributed and the atomic percent of Zn element increased from 5.16% to 22.50%.

On the other hand, the digital photographs and SEM images of pristine poplar wood are provided, as shown in Figure S3, and the morphology of fir wood as a kind of coniferous wood was also observed for comparison to illustrate the difference in emulsification performance. The result illustrates that both fir wood and poplar have interesting microstructures consisting of numerous long and arrayed lumens interconnected by pits and ray cells along the growth direction, forming oriented interpenetrating capillary networks. For poplar wood, two kinds of cellular structure could be observed. Vessels have larger lumen diameters of about 80–100 μm, while those of fibers are about 10–20 μm. Compared with poplar, fir wood has a more uniform size of tracheid lumen, of about 30 μm. The light microscope images from Figure S4 further confirm that the wood has highly aligned cell lumens and interconnected pit/ray cell structures. Pits and wood rays play a crucial role in facilitating the horizontal transport of nutrients within trees, which could facilitate the mixing process of microfluids along the growth direction during membrane emulsification.

**Figure 6:**
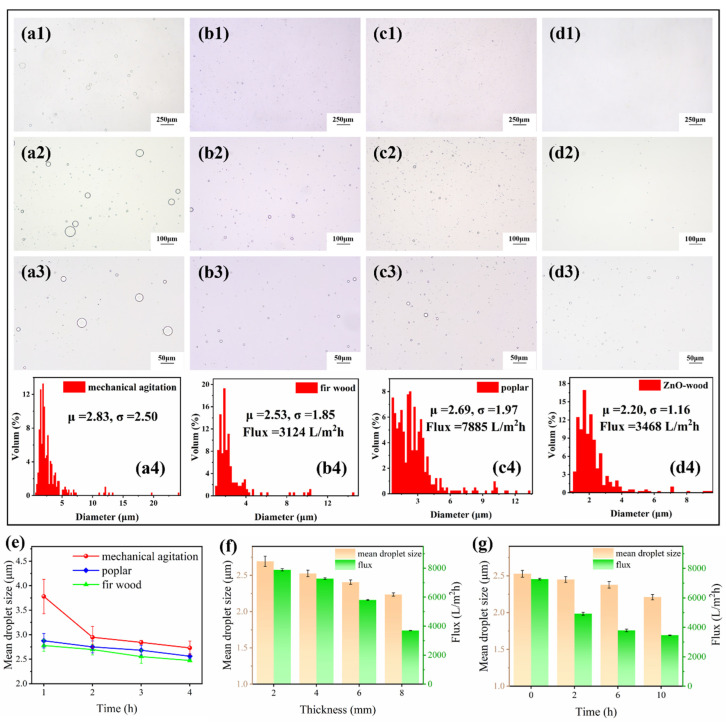
Optical microscope images and droplet size distributions of oil-in-water emulsions (**a1**–**a4**) with mechanical agitation process. (**b1**–**b4**) Fir wood, (**c1**–**c4**) poplar and (**d1**–**d4**) ZnO-wood; (**e**) effect of treatment time on the droplet size; (**f**) effect of pristine poplar thickness on droplet size and flux; (**g**) droplet size and flux of oil-in-water emulsion from ZnO-wood membrane prepared by various hydrothermal times.

To demonstrate the advantages of the ZnO-wood membrane, it was employed to produce an oil-in-water emulsification, and both pristine poplar and fir wood are also involved for comparison (Figure 6). The membrane was settled in a homemade emulsification unit with an air pressure of 0.1 bar for membrane emulsification. As mechanical agitation is a common method to prepare oil-in-water emulsions, its emulsification performance was also compared. It was found that the droplet size of emulsions treated with mechanical agitation is heterogeneous and that many large-size droplets exist. However, there are no large-size droplets in the emulsion prepared by the membrane emulsification process, which displays a narrow size distribution. Moreover, the emulsion from the ZnO-wood membrane exhibits smaller droplet sizes and a narrower size distribution than pristine poplar and fir wood. The result also shows that, although poplar and fir wood have a similar emulsion size, poplar (78,850 L·m^−2^·h^−1^·bar^−1^) has a much higher flux than fir wood (31,240 L·m^−2^·h^−1^·bar^−1^). Hence, poplar is a suitable candidate for designing high-throughput membrane emulsifications. It was also found that mechanical agitation time affects the emulsion quality: the longer the time, the smaller the droplet size. Moreover, the greater thickness of poplar decreased the size of the emulsion while sacrificing treatment efficiency. In addition, the emulsifying property of the ZnO-wood membrane with various hydrothermal time was studied. With increasing hydrothermal time, the droplet size of ZnO-wood became smaller and had a lower standard deviation, which was further confirmed by the statistical result, as shown in Figure S5. However, the emulsion flux declined gradually, which may be due to the raised content of ZnO nanoparticles shrinking the size of the wood capillary channel. Even so, ZnO-wood prepared by 10 h of hydrothermal time maintained an impressive emulsion flux of 34,680 L·m^−2^·h^−1^·bar^−1^. The droplet size and distributions of oil-in-water emulsions were also accurately characterized by the Malvern Zetasizer, as shown in Figure S6. The result shows that the sizes of droplet characterized by the Malvern Zetasizer are much smaller than the results of the statistical analysis of the optical microscope images. This further indicates that the droplets obtained from the ZnO-wood membrane have a minimum size at the nanoscale (average droplet size of about 400 nm). At the same time, they have much more uniform size distribution, mainly concentrated around 10 nm, compared with the mechanical agitation and wood membrane emulsification processes. Compared with other membrane materials in the references [56,57,58], the ZnO-wood membrane in this work has smaller and narrower droplet size with higher fluxes. Additionally, the membrane emulsification fluxes were recorded for three consecutive membrane emulsification cycles, as shown in Figure S7. It was found that the ZnO-wood membrane has a relatively stable flux, suggesting a decent stability during continuous emulsification. These results indicate that the membranes have excellent emulsification performance.

**Figure 7:**
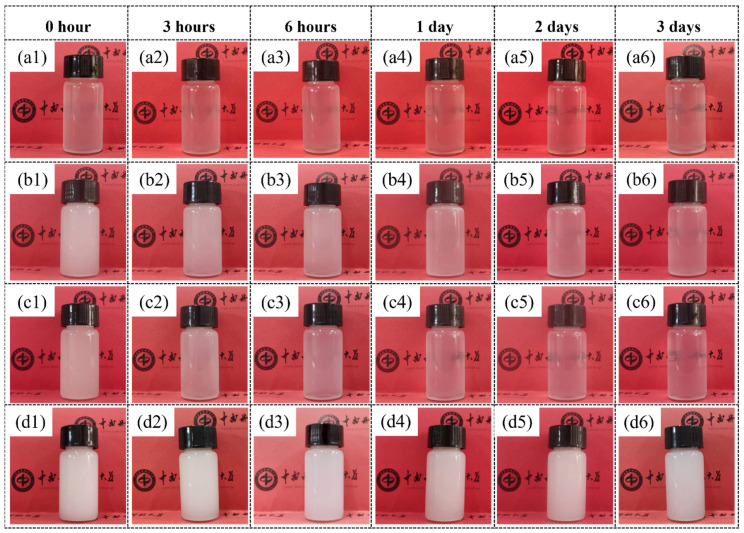
The stability of oil-in-water emulsions formed by: (**a1**–**a6**) mechanical agitation; (**b1**–**b6**) fir wood membrane emulsification process; (**c1**–**c6**) poplar membrane emulsification process; (**d1**–**d6**) ZnO-wood membrane emulsification process.

In addition, the stability of these emulsions made from the ZnO-wood membrane are also illustrated by a standing test. Figure S5 shows that the oil-in-water emulsion by ZnO-wood membrane emulsification remained stable after 3 days of deposition and that the ZnO-wood membrane prepared by 10 h of hydrothermal time exhibits the best emulsion stability. Comparing the emulsion stability of mechanical agitation and pristine wood, Figure 7 shows that the ZnO-wood membrane displays incomparable emulsion stability. The emulsion from mechanical agitation has the lowest concentration and poor stability. This also indicates that both poplar and fir wood membranes can produce higher emulsion concentrations than mechanical agitation, but their emulsion stability is inadequate.

**Figure 8:**
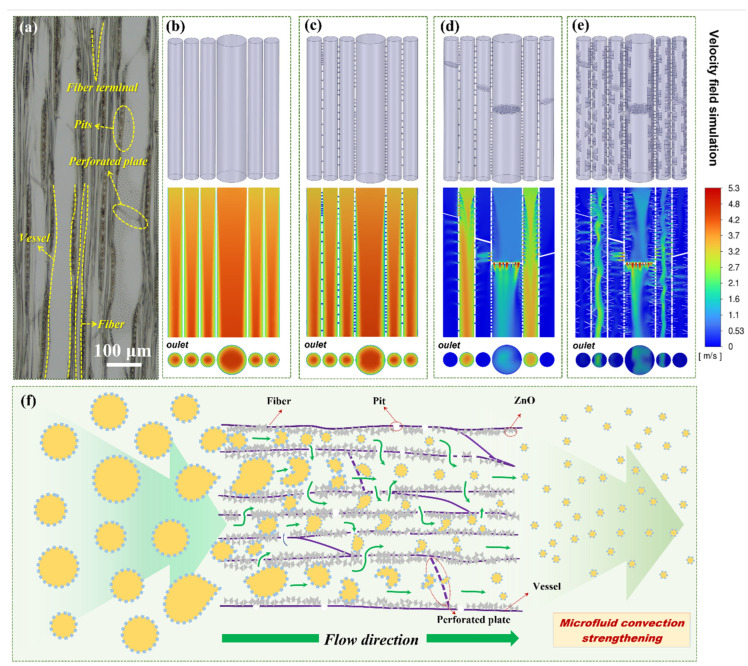
(**a**) Optical microscope image of poplar wood, showing four separate geometries and their velocity field distribution from ANSYS Fluent simulation. (**b**) Oriented capillary array without any connections and perforated plates/fiber terminals, (**c**) oriented capillary array with connection via pits on wall, (**d**) oriented capillary array with connections and perforated plates/fiber terminals, (**e**) oriented capillary array with connections and perforated plate/fiber terminals (rough surface structure); (**f**) schematic of emulsion process analysis in oriented interpenetrating capillary network of ZnO-wood membrane.

In order to further understand the mechanism of emulsification enhancement, the influence of wood cellular structure and its surface engineering on microfluid transport were investigated by ANSYS Fluent simulation version 2022 R1). The inlet pressure of the microchannel was set at 0.1 bar plus one atmospheric pressure, simulating experimental conditions, while the outlet pressure was set at atmospheric pressure. As shown in Figure 8a, the optical microscope image indicates that poplar wood consists of numerous long and arrayed lumens that include larger-size vessels and smaller fiber cellular structures. Adjacent cell lumens are interconnected by pits and ray cells in transverse direction. Perforated plates connect vessels along the growth direction. Based on the structural features of wood, four separate geometries were established to demonstrate the effect of the ZnO-wood structure on microfluidic mixing and mass transfer in the wood capillary channels, in which surface engineering by ZnO nanoparticles was also contained. The four separate geometries are as follows: oriented capillary arrays without any connections and perforated plates/fiber terminals (Figure 8b), oriented capillary arrays with connections through pits in the wall (Figure 8c), oriented capillary arrays with connections and perforated plates/fiber terminals (Figure 8d), and oriented capillary arrays with connections and perforated plates/fiber terminals as well as rough channel surfaces (Figure 8e). The simulations were performed on wood channels with these four separate geometries, and their velocity field distributions from the ANSYS Fluent simulation are also provided. The results illustrate that there is hardly any mixed convection in straight capillary arrays without any connections. The microfluids along the channels display typical characteristics of a laminar flow regime [59]. In the context of a micrometer-scale channel, it leads to low Reynolds numbers. At low Reynolds numbers, viscous forces dominate over inertial forces, resulting in laminar flow. The flow is characterized by the absence of turbulence, vortices, or irregular mixing. Each microfluid unit follows a well-defined path without crossing or mixing with neighboring units, even though these channels are interconnected by wall pits. Changing the geometric structure of the microchannel is an effective way to improve the mixing efficiency for membrane emulsification [60]. As shown in Figure 8d, the mixed convection is significantly enhanced in the presence of perforated plates/fiber terminals. Adjacent microfluids are interpenetrated, forming mixed convection. The simulated result from Figure 8e also indicates that the mixing efficiencies are further greatly improved by designing convex grooves in these channels. It can be concluded that the rough channel surface formed by the unique ZnO layer in the wood capillaries is able to further intensify the microfluid mixing process.

Hence, the mechanism of emulsification enhancement in an oriented interpenetrating capillary network for a ZnO-wood membrane was proposed, and a schematic of emulsion process analysis is shown in Figure 8f. The ZnO-wood membrane possesses an oriented interpenetrating capillary network composed of highly aligned and interconnected wood cell lumens. When water containing oil permeates into the ZnO-wood membrane, the wall of the cell lumen is first wetted by water and restrains the formation of a continuous oil film due to excellent lipophobicity and hydrophilicity. Large oil droplets in microchannels are squeezed and broken down into smaller droplets continuously along long cell lumens by convective mixing until they are extruded from the membrane. The well-designed honeycomb porous ZnO layer boosts convective mixing through shrinking microchannel size and increasing wall roughness. Meanwhile, oil droplets are squeezed repeatedly across pits and perforated plates, causing the dispersed phase to generate smaller-size oil droplets with narrower size distribution. In addition, these oriented and straight capillary arrays ensure high throughput for emulsion production. Therefore, high-quality emulsions with high throughput can be achieved simultaneously by the designed ZnO-wood membrane.

## 4. Conclusions

In summary, wood with a novel honeycomb porous ZnO layer has been successfully fabricated, and the hydrothermal growth time has a significant influence on the morphology of the ZnO layer. This demonstrates that an oriented interpenetrating capillary network from wood decorated with a unique ZnO layer can function as an excellent membrane emulsion material. The designed membrane is capable of producing high-quality emulsions with high throughput simultaneously, which is attributed to the synergistic effect of the unique channel structure of the wood and evenly distributed ZnO layer particles. ZnO-wood prepared by 10 h hydrothermal time maintains an impressive emulsion flux of 34,680 L·m^−2^·h^−1^·bar^−1^ with a mean droplet size of about 400 nm. ANSYS Fluent simulation further proves the mechanism of enhanced emulsification; that is, the convection process of the microfluid within the membrane is significantly enhanced. The proposed wood-based emulsification membrane is cost-effective and high-performance, scalable, and enables potential industrial-scale emulsion processes. In our follow-up work, the designed emulsifying membrane will attempt to produce micro-/nanoparticles with tailored properties. Such a productive capability could open up a whole new field for functional applications of wood.

## Data Availability

Data can be obtained from the corresponding author upon a reasonable request (due to privacy).

## Supplementary Materials

The following supporting information can be downloaded at: https://www.mdpi.com/article/10.3390/ma17092113/s1, Figure S1: (a) The thermogravimetric degradation of ZnO-wood in an oxygen atmosphere; (b) Digital photographs of ZnO-wood before and after heat treatment and N-Methylpyrrolidone soaking, respectively; Figure S2: SEM images of ZnO-wood with various hydrothermal time: (a,b) 2 h; (c,d) 6 h; (e,f) 10 h; the element mappings and EDS spectrum images of ZnO-wood with (g) 2 h, (h) 6 h and (i) 10 h hydrothermal time; Figure S3: Digital photographs and SEM images of (a1–a7) fir wood and (b1–b7) poplar; Figure S4: The light microscope images of (a) fir and (b) poplar wood along the growth direction; Figure S5: The droplet size distributions and digital images of oil-in-water emulsions from ZnO-wood membrane prepared by different hydrothermal times: (a1–a7) 2 h; (b1–b7) 6 h; (c1–c7) 10 h; (1) digital photograph; (2) optical microscope image; (3) droplet size distribution; Figure S6: The droplet sizes and their distributions characterized by the Malvern Zetasizer: (a1–a3) three parallel experiments obtained by mechanical agitation; (b1–b3) three parallel experiments obtained from wood membrane; (c1–c3) three parallel experiments obtained from ZnO-wood membrane; (d) their mean drop-size analysis. Figure S7. The membrane emulsification fluxes during three consecutive membrane emulsification cycles.
