# Supplementary material for: Oriented Interpenetrating Capillary Network with Surface Engineering by Porous ZnO from Wood for Membrane Emulsification
Source: Materials (Basel). 2024 Apr 30;17(9):2113. doi: 10.3390/ma17092113 (PMC11084715; doi:10.3390/ma17092113)
Supplement: Supplementary file 1 [file materials-17-02113-s001.zip › materials-2969021-supplementary.pdf]

Supplementary Materials

Oriented Interpenetrating Capillary Network with Surface Engineering by Porous ZnO from Wood for Membrane Emulsification

Yaodong Chen [1], Xiaolin Liu [2], Gonggang Liu [1,*], Shanshan Chang [1] and Jinbo Hu [1,*]

1 Hunan Province Key Laboratory of Materials Surface & Interface Science and Technology, College of Materials Science and Engineering, Central South University of Forestry and Technology, Changsha 410004, China; cyd222610@163.com (Y.C.); changelxy@hotmail.com (S.C.)

2 Hunan Lintec Co., Ltd., Changsha 410600, China; liuxiaolin@lintec-x.com

* Correspondence: liugonggang@csuft.edu.cn (G.L.); jinbo.hu@csuft.edu.cn (J.H.)

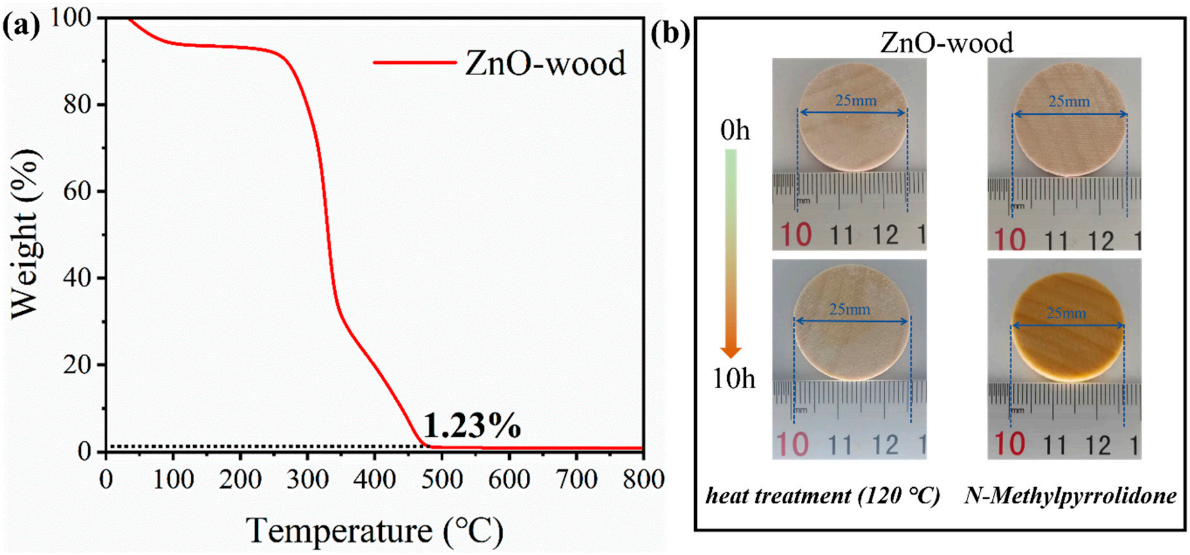

Figure S1. (a) The thermogravimetric degradation of ZnO-wood in an oxygen atmosphere; (b) Digital photographs of ZnO-wood before and after heat treatment and N-Methylpyrrolidone soaking, respectively.

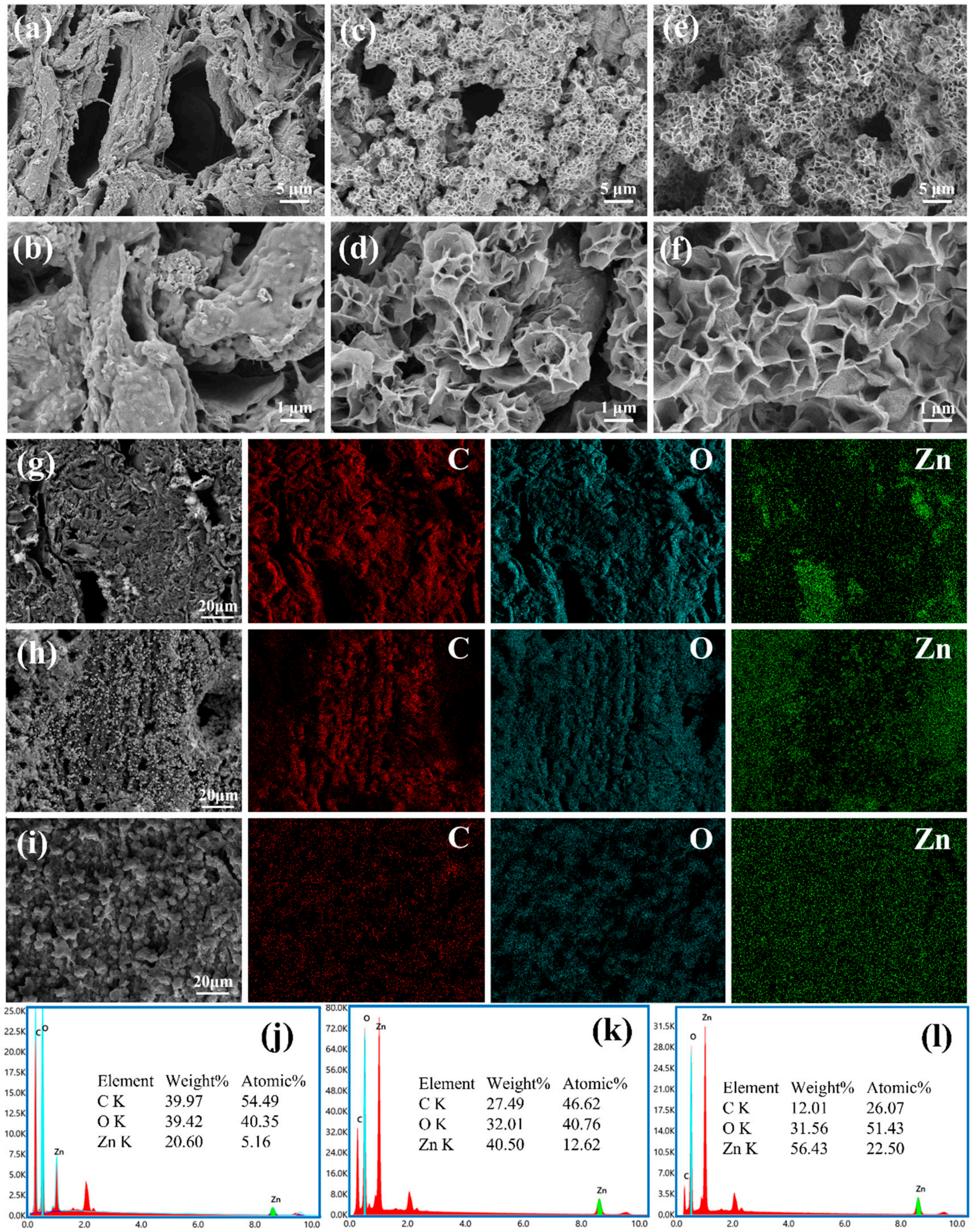

Figure S2. SEM images of ZnO-wood with various hydrothermal time: (a, b) 2h; (c, d) 6h; (e, f) 10h; the element mappings and EDS spectrum images of ZnO-wood with (g, j) 2h, (h, k) 6h and (i, l) 10h hydrothermal time.

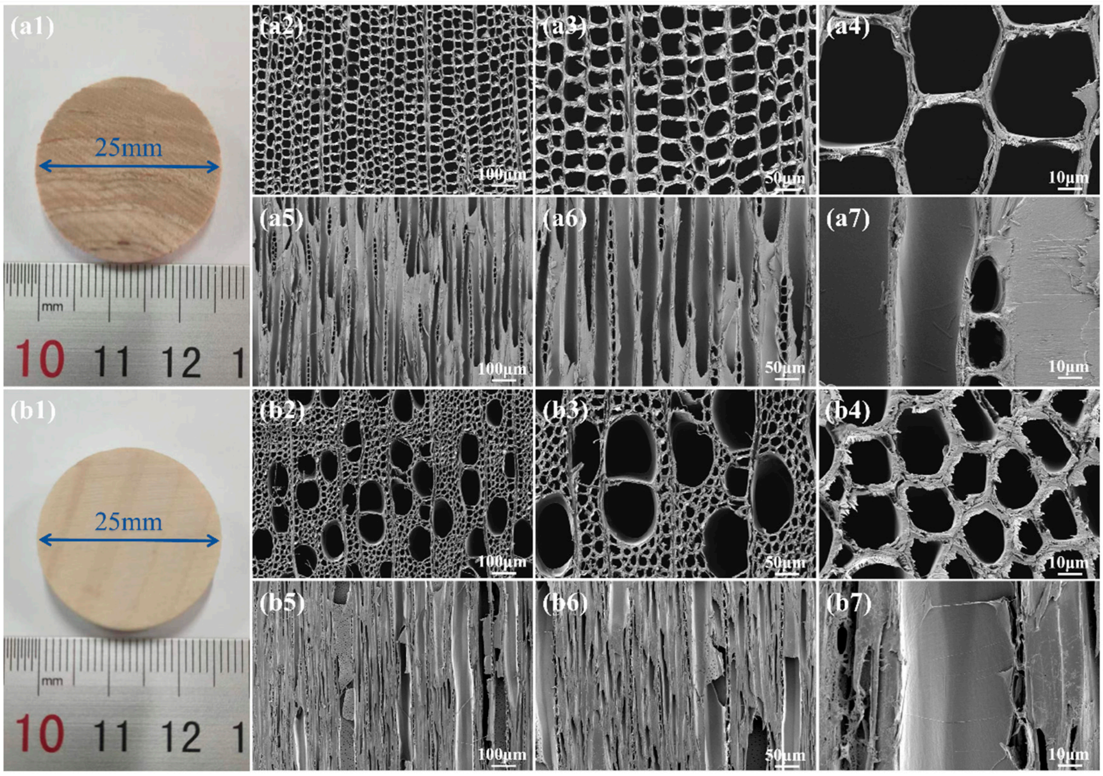

Figure S3. Digital photographs and SEM images of (a1-a7) fir wood and (b1-b7) poplar.

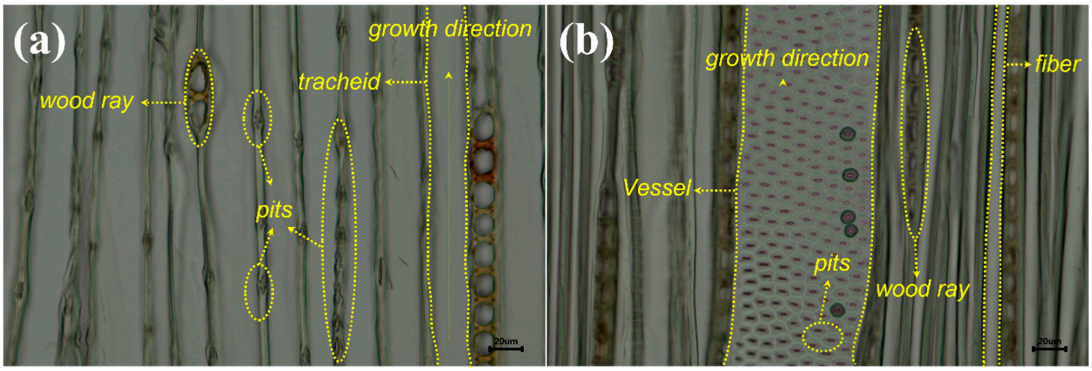

Figure S4. The light microscope images of (a) fir and (b) poplar wood along the growth direction

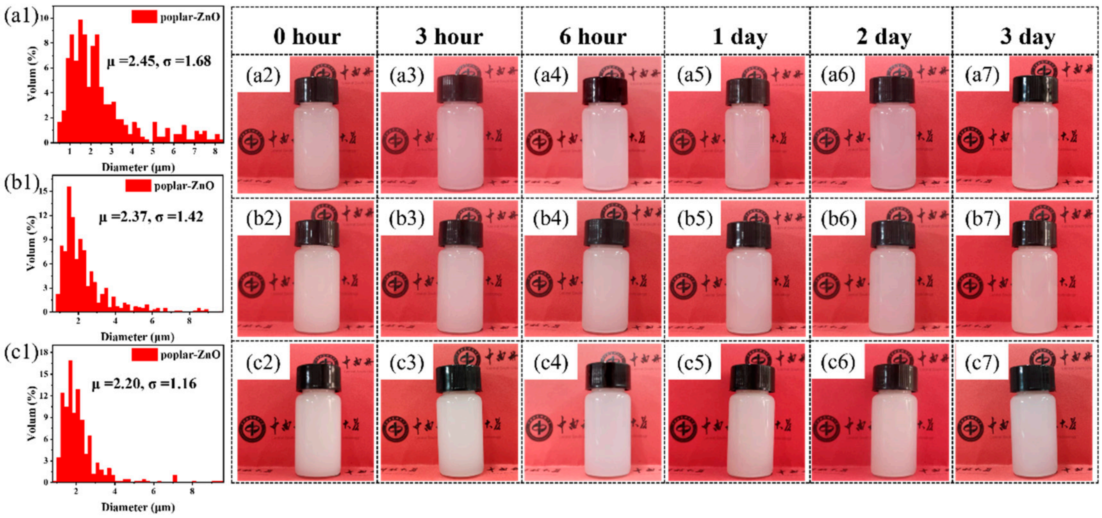

Figure S5. The droplet size distributions and digital images of oil in water emulsions from ZnO-wood membrane prepared by different hydrothermal time: (a1-a7) 2h; (b1-b7) 6h; (c1-c7) 10h; (1) digital photograph; (2) optical microscope image; (3) droplet size distribution.

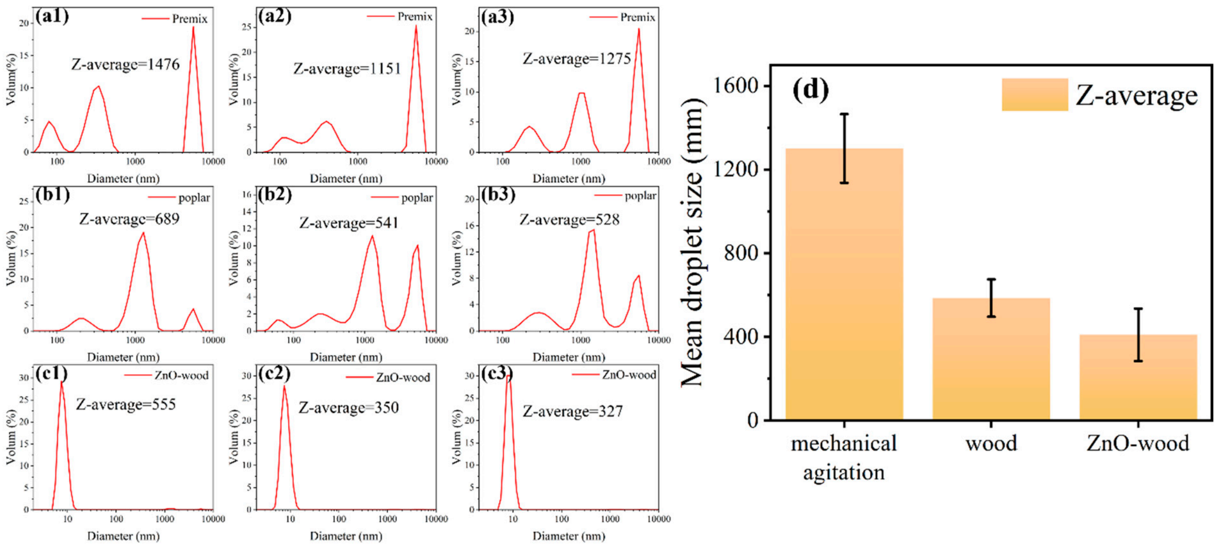

Figure S6. The droplet sizes and their distributions characterized by the Malvern Zetasizer: (a1-a3) three parallel experiments obtained from mechanical agitation; (b1-b3) three parallel experiments obtained from poplar wood membrane; (c1-c3) three parallel experiments obtained from ZnO-wood membrane; (d) their mean drop size analysis.

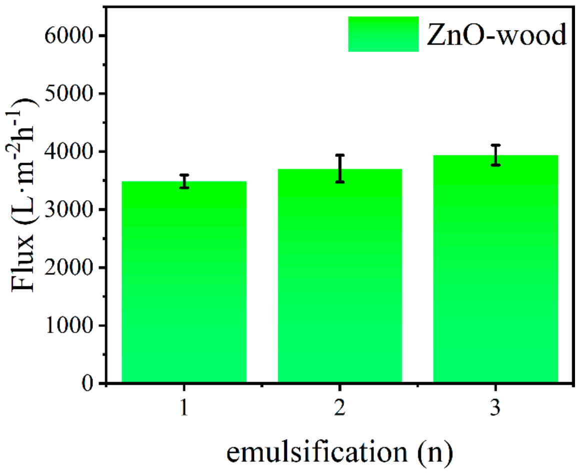

Figure S7. The membrane emulsification fluxes during three consecutive membrane emulsification cycles.
